# Delayed Auditory Brainstem Responses in Prelingually Deaf and Late-Implanted Cochlear Implant Users

**DOI:** 10.1007/s10162-015-0532-x

**Published:** 2015-07-11

**Authors:** Marc J. W. Lammers, Ruben H. M. van Eijl, Gijsbert A. van Zanten, Huib Versnel, Wilko Grolman

**Affiliations:** Department of Otorhinolaryngology and Head & Neck Surgery, University Medical Center Utrecht, Room G.02.531, P.O. Box 85500, 3508 GA Utrecht, The Netherlands; Brain Center Rudolf Magnus, University Medical Center Utrecht, Utrecht, The Netherlands

**Keywords:** auditory brainstem response, cochlear implant, maturation, humans, prelingual deaf, hearing loss, deafness

## Abstract

Neurophysiological studies in animals and humans suggest that severe hearing loss during early development impairs the maturation of the auditory brainstem. To date, studies in humans have mainly focused on the neural activation of the auditory brainstem in children treated with a cochlear implant (CI), but little is known about the pattern of activation in adult CI users with early onset of deafness (prelingual, before the age of 2 years). In this study, we compare auditory brainstem activation in prelingually deaf and late-implanted adult CI users to that in postlingually deaf CI users. Electrically evoked auditory brainstem responses (eABRs) were recorded by monopolar stimulation, separately using a middle and an apical electrode of the CI. Comparison of the eABR latencies revealed that wave V was significantly delayed in the prelingually deaf CI users on both electrode locations. Accordingly, when the apical electrode was stimulated, the III–V interwave interval was significantly longer in the prelingually deaf group. These findings suggest a slower neural conduction in the auditory brainstem, probably caused by impairment of maturation during the long duration of severe hearing loss in infancy. Shorter wave V latencies, reflecting a more mature brainstem, appeared to be a predictor for better speech perception.

## INTRODUCTION

The duration of auditory deprivation before cochlear implantation is an important predictor for hearing with a cochlear implant (CI). Whereas patients with late onset of deafness can obtain good speech perception in quiet, the hearing performance of patients with long-term early-onset deafness is generally poor (Teoh et al. 2004; Lammers et al. 2015). The large difference might be caused by an impaired development of their auditory pathway in combination with cross-modal changes during a prolonged period of auditory deprivation (Doucet et al. 2006; Lee et al. 2007; Kral and O’Donoghue 2010; Kral and Sharma 2012; Lammers et al. 2015). Recently, we demonstrated that prelingually deaf and late-implanted CI users display relatively early and large N1 peaks of the cortical auditory-evoked potential (Lammers et al. 2015). This altered cortical activity raises the question regarding the extent to which the subcortical pathway, particularly the auditory brainstem, is affected in prelingually deaf CI users.

It is well documented that after birth, auditory brainstem response (ABR) wave latencies decrease and reach adult levels around the age of 2–3 years (Inagaki et al. 1987; Eggermont and Salamy 1988). This decrease is slower for wave V than for early waves and is hypothesized to result from increasing myelination and/or synaptic efficacy within the auditory brainstem since these developments lead to faster axonal conduction and synaptic transmission (Eggermont and Salamy 1988; Moore et al. 1995; Thai-Van et al. 2007). Long periods of deafness affect the subcortical pathway, resulting in gradual spiral ganglion cell degeneration (Spoendlin 1975; Versnel et al. 2007), and a volume reduction of the cochlear nucleus and its cells (Moore 1990; Leake et al. 2008; Ryugo et al. 2010). On the other hand, electrically evoked ABRs (eABRs) in congenitally deaf cats demonstrate latencies decreasing with age, similarly to normal-hearing cats (Tillein et al. 2012). This suggests that auditory brainstem structures and pathways develop even in the absence of auditory stimulation.

In humans, development of the auditory brainstem following deafness has been studied by recording eABRs in children with CI (Gordon et al. 2006, 2008; Thai-Van et al. 2007; Sparreboom et al. 2010). These studies demonstrated that in children with early-onset deafness, eABR wave latencies decrease after implantation, irrespective of age at implantation, like they do in normal-hearing children. On the contrary, in bilaterally implanted children, when a response is evoked using a second CI implanted much later than the first, the wave V latency is longer than the responses evoked by the first CI (Gordon et al. 2008; Sparreboom et al. 2010). This suggests impaired maturation of the auditory brainstem of the later implanted ear (Gordon et al. 2008; Sparreboom et al. 2010), or altered neuronal connections induced by the period of unilateral hearing with the first CI.

Whereas above-mentioned studies were performed in children and in animal models, we address the effect of early deafness on the auditory brainstem in adults. We compare eABRs in prelingually deaf subjects who had little or no auditory stimulation for more than 20 years to eABRs in postlingually deaf CI users. According to various studies (Eggermont and Salamy 1988; Moore et al. 1995; Thai-Van et al. 2007; Leake et al. 2008; Ryugo et al. 2010), myelination and synapses in the brainstem of the postlingual group should have developed normally because of sufficient auditory input during childhood. In contrast, we expect the coarse structures of the brainstem to develop in the prelingual group but sensory-driven maturation to be impaired reducing axonal myelination and synaptic efficacy among others. Any effect by auditory stimulation after cochlear implantation could only have occurred in the adult system and is expected to be negligible. Therefore, we hypothesize typical eABR waveforms in both groups, but longer wave V latencies in the prelingually deaf.

## METHODS

### Participants

All adult users of a Cochlear® CI who visited the outpatient clinic from December 2011 to December 2012 were consented to participate in a study which included eABR and cortical auditory-evoked potential (CAEP) recordings. Twenty-three adults, with at least 6 months experience with their CI, agreed to participate in this study. In 20 subjects, eABRs could be recorded, while in the remaining three postlingual subjects, no clear eABRs could be evoked due to electrical artifacts contaminating the waveforms. Their data were therefore not included in the analyses. Due to time constraints, in one subject eABRs without CAEPs were obtained (post 1). Prelingually deaf subjects were selected based on the following criteria: onset of severe to profound binaural hearing loss before the age of 2 years (based on medical charts including diagnostic audiometry and self-reported patient information) and insufficient residual hearing during childhood for normal speech and language development. Based on these criteria, the diagnosis of prelingual deafness was confirmed by the multidisciplinary CI team prior to implantation. Eleven adults met these criteria and were thus labeled as prelingually deaf. Nine adults became deaf during adolescence or adulthood (>15 years of age) and were categorized as postlingually deaf. All participants were users of Nucleus multi-channel CIs, and in all subjects a full insertion of the electrode array was achieved. Table 1 summarizes detailed patient characteristics. The data of the CAEPs (recorded in 22 subjects, including all 11 prelingual subjects and 8 postlingual subjects enrolled in this study) have been reported in a separate paper (Lammers et al. 2015).

**TABLE 1:** Subject demographics

Subject	Group	Side CI	Etiology	Age at test (years)	Age at onset deafness (years)	Age at CI (years)	Implant experience (years)	Pre-op CVC score (%)	Post-op CVC score (%)^a^	Primary mode of communication (pre-operative)
Pre 1	Prelingual	Right	Meningitis	23	0.5	21	1.7	0	22 (3)	Lip reading
Pre 2	Prelingual	Right	Congenital	38	0	37	0.6	0	0 (1)	Lip reading and sign language
Pre 3	Prelingual	Left	Rubella	56	0.3	54	2.5	0	82 (5)	Lip reading
Pre 4	Prelingual	Left	Varicella	55	2	55	0.6	0	26 (5)	Lip reading and sign language
Pre 5	Prelingual	Left	Rubella	47	0	46	1.5	48	66 (4)	Oral and lip reading
Pre 6	Prelingual	Left	Meningitis	55	0.8	49	5.7	12	18 (0)	Lip reading and sign language
Pre 7	Prelingual	Left	Meningitis	47	0.8	41	5.7	0	28 (5)	Lip reading and sign language
Pre 8	Prelingual	Right	Congenital	43	0	40	2.9	0	0 (9)	Lip reading and sign language
Pre 9	Prelingual	Left	Unknown	31	0	27	3.2	0	15 (8)	Lip reading and sign language
Pre 10	Prelingual	Right	Rubella	42	0	41	1.4	0	0 (1)	Lip reading and sign language
Pre 11	Prelingual	Left	Unknown	36	0	27	8.9	28	77 (0)	Lip reading and sign language
Mean				43	0	40	3.2	8	30	
Post 1	Postlingual	Left	Progressive	75	68	68	6.5	0	78 (0)	Oral
Post 2	Postlingual	Right	Otitis media	80	64	75	5.3	37	89 (2)	Oral
Post 3	Postlingual	Right	Otitis media	58	27	43	14.4	–	60 (3)	Oral and lip reading
Post 4	Postlingual	Right	Unknown	73	50	59	13.8	–	72 (0)	Oral
Post 5	Postlingual	Right	Progressive	57	29	50	6.9	28	95 (7)	Oral and lip reading
Post 6	Postlingual	Left	Trauma	22	16	16	6.5	0	90 (0)	Oral
Post 8	Postlingual	Right	Progressive	59	43	45	13.3	25	85 (3)	Oral and lip reading
Post 9	Postlingual	Right	Progressive	53	40	41	11.9	11	84 (8)	Oral
Post 11	Postlingual	Left	Meningitis	33	15	25	8.2	0	97 (0)	Oral
Mean				57	39	47	9.6	14	83	

– not performed

^a^Months between eABR recording and CVC measures are displayed between brackets in the post-op CVC score column

### Speech Perception

Speech perception scores were obtained using the Dutch Society of Audiology standard consonant-vowel-consonant (CVC) word list at 65 dB SPL (Versfeld et al. 2000). In this open-set test, only auditory cues were available. Speech perception was scored based on the number of phonemes correctly identified. For each subject, the most recent scores prior to the evoked potential recordings were used (time intervals between 0 and 9 months).

### Procedure and Stimuli

Participants were seated in a comfortable reclining chair in an electrically shielded, sound attenuated booth and were asked to keep their eyes closed and minimize movements. The electric stimulus consisted of a biphasic pulse, with a phase width of 25 μs and an inter-phase gap of 8 or 58 μs. A monopolar stimulation electrode configuration was applied, and two positions of the active electrode were used: at the apical end of the array (typically electrode no. 20) and a central position (typically electrode no. 11). A basal electrode was also used (as it was for CAEPs, Lammers et al. 2015), but due to stimulation artifacts, the signal-to-noise ratio of the eABRs was too low to obtain reliable and reproducible waveforms in several patients. Stimuli were generated using the Cochlear Custom Sound EP 3.1 software and presented at a rate of 35 Hz at the individual’s maximum comfortable loudness level (C-level). For each subject, 1,500 accepted sweeps were averaged. Multiple additional replications at the same level and lower stimulus levels were recorded to confirm the response. Stimulation levels were decreased until wave V could not be distinguished anymore. For data analyses, only the response obtained at C-level was used for each subject.

### Evoked Potential Recording

Responses were recorded by Ag/AgCl electrodes placed according to the 10–20 system at Cz and Fz using a Medelec Synergy T-10 Evoked Potential system. The ground was placed on the forehead and the contralateral mastoid was used as reference. Recordings were filtered from 100 Hz to 5 kHz and recorded with a sampling rate of 20 kHz. Sweeps containing signals of >50 μV at any electrode were rejected and not included in the average signal. Electrode impedances were kept below 5 kΩ.

### Data Analyses

Averaged eABR data were analyzed using custom scripts in MATLAB (version 7.11.0, Mathworks). For each subject, the analysis of the wave III and wave V latencies and the III–V interval was based on the eABRs obtained at maximum comfort level. Waves III and V were manually identified by two authors (MJWL and RHMvE) independently. Disagreements were resolved by discussion.

Statistical analyses were completed using SPSS version 22.0 software. Repeated measures ANOVAs with the two different intracochlear stimulus locations (i.e., middle and apical) as within-subjects factor and group (i.e., prelingual or postlingual) as between-subjects factor were used. Significant main effects and interactions (*p* < 0.05) were followed with Bonferroni post hoc tests, and the Greenhouse–Geisser correction was applied to compensate for violations of the sphericity assumption. Group differences in peak latencies were calculated with unpaired, two-tailed *t* tests or the Mann–Whitney test for independent samples. Stepwise multiple linear regression analyses were performed to evaluate the influence of other variables on wave latencies. Linear regression analyses were performed to evaluate the relationship between speech perception and eABR latencies.

## RESULTS

### Patient Demographics and Speech Perception

**FIG. 1:**
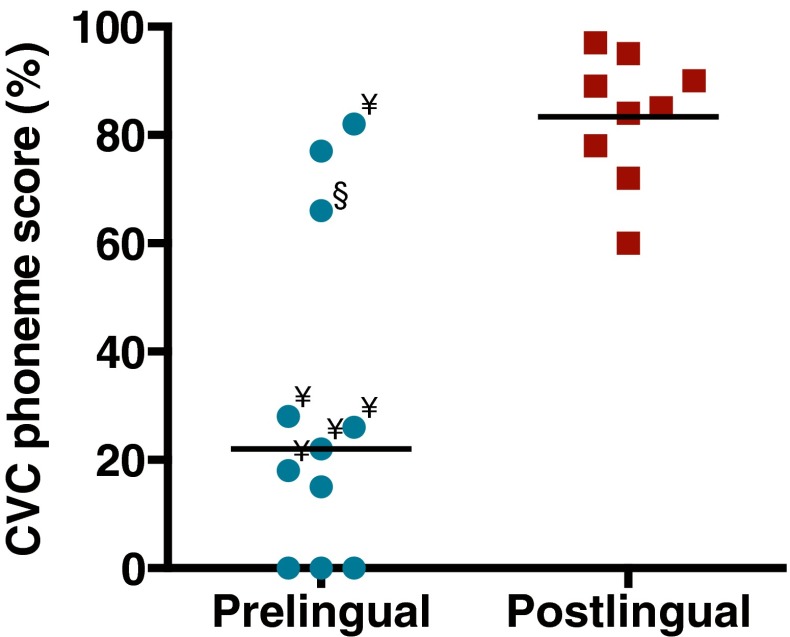
Consonant-vowel-consonant (CVC) phoneme scores of the prelingual and postlingual group taken prior to the evoked potential recordings. Each *dot* represents an individual subject. The *horizontal lines* represent the median scores (prelingual group, 22 %; postlingual group, 85 %). Subjects with hearing experience (pre 1, 3, 4, 6, and 7) are marked with *yen sign*; subject pre 5, who used both visual speech cues and oral communication preoperatively, is marked with *section sign*. This plot is a partial replot of Figure 1 in Lammers et al. (2015); all 11 prelingual data points and 8 of 11 postlingual data points of that paper are plotted here.

The monosyllabic open-set speech perception scores varied widely among the patients, ranging from 0 to 82 % in the prelingual group and from 60 to 97 % in the postlingual group (Fig. 1). Median speech perception scores were significantly higher in the postlingual group (median 85 % correct) than in the prelingual group (22 % correct; Mann–Whitney *U* = 6.0, *P* < 0.0001). Patient demographics are presented in Table 1. The prelingual group and the postlingual group differed significantly in implant experience (mean difference: 6.5 years; unpaired, two-tailed *t* test, *t*_*18*_ *=* 4.6, *P* < 0.001). Age at implantation did not differ significantly (unpaired, two-tailed *t* test, *t*_*18*_ *=* 1.1, *P* = 0.305), neither did age at testing (unpaired, two-tailed *t* test, *t*_*18*_ *=* 2.0, *P* = 0.057). Preoperative CVC phoneme scores were not different between the two groups.

### eABR Waveforms in the Pre- and Postlingual Groups

**FIG. 2:**
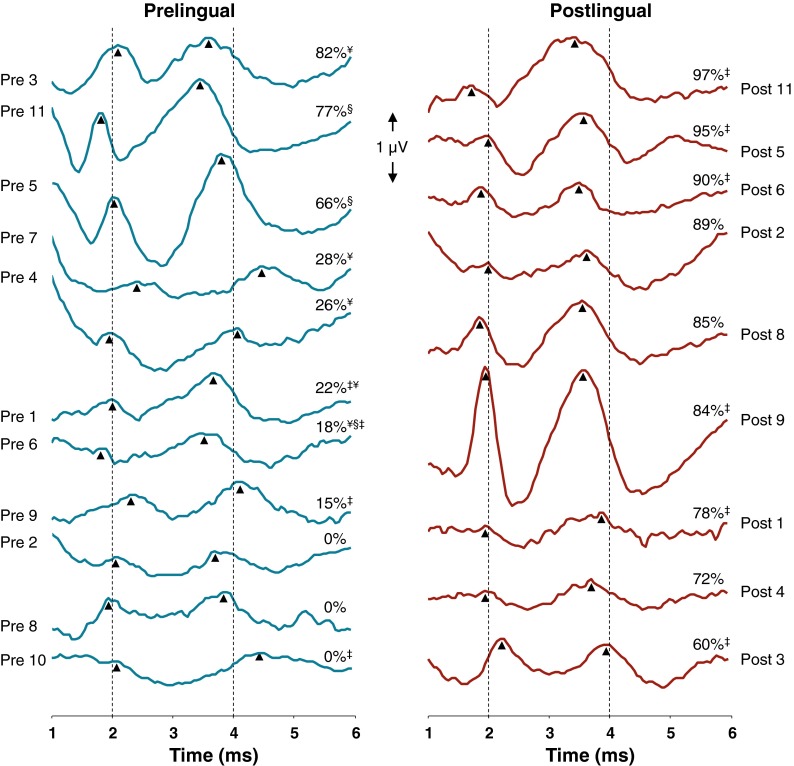
Individual eABR waveforms evoked at an apical electrode. In cases in which large stimulus artifacts partially obscured the measurements recorded on Cz, waveforms measured at electrode Fz are presented here (denoted with *double dagger sign*). Waveforms were corrected for stimulus artifact by fitting a first-order polynomial and subtracting it from the signal. In almost all subjects in both groups, wave III (indicated with *first upward arrowhead*) and wave V (indicated with *second upward arrowhead*) could be identified. The *vertical lines* drawn near the peak latencies of waves III and V are shown to facilitate comparisons between subjects. At the right side of the individual waveforms, the CVC phoneme scores are presented. In the outer margins of the figure the patient numbers are presented. Subjects with hearing experience (pre 1, 3, 4, 6, and 7) are marked with *yen sign*; subjects who had a preoperative CVC phoneme score higher than 0 % correct (pre 5, 6 and 11) are marked with *section sign*.

In all nine postlingual subjects, reproducible eABR waveforms could be obtained on both electrodes. In 9 out of 11 prelingual subjects, eABR waveforms could be obtained on both electrodes, whereas in two subjects no clear waves III and V could be identified on the middle electrode. Figure 2 shows the individual eABR waveforms evoked at an apical electrode (typically electrode no. 20) for prelingual (left) and postlingual (right) subjects in order of CVC score. The waveforms tended to be relatively small for the prelingual subjects with poor speech perception. Depending on factors such as location of recording and stimulation electrodes, head size, and skull thickness, eABR wave amplitudes were highly variable among subjects and thus less reliable for group comparisons. Therefore, only wave latencies were considered for group comparisons.

### Wave Latencies

**FIG. 3:**
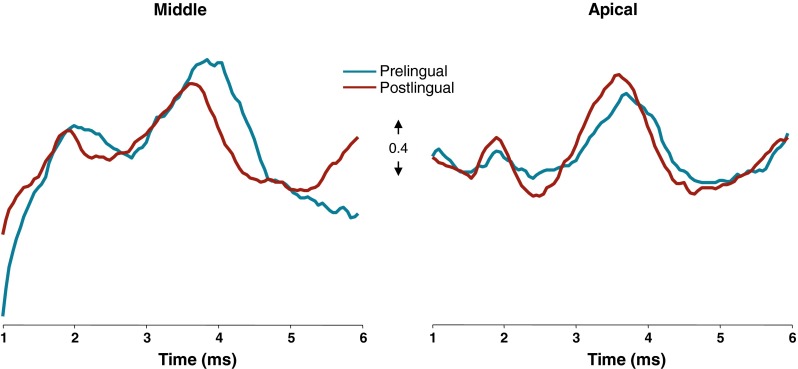
Grand average eABR waveforms measured at Cz for all subjects in both groups, presented for the two stimulation electrode locations separately. The *blue and red traces* represent the waveforms of the prelingual and postlingual groups, respectively. Waveforms were first corrected for stimulus artifact by fitting a first-order polynomial and subtracting it from the signal and then they were normalized by dividing the signal by the difference in amplitude between the top of wave V and its preceding trough.

**FIG. 4:**
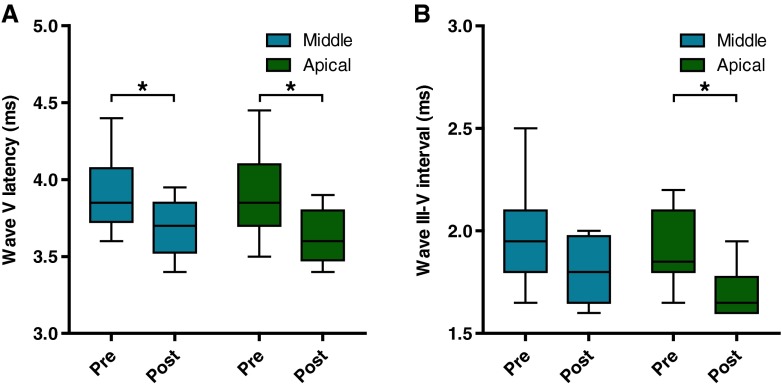
Wave V latencies (*A*) and III–V interwave intervals (*B*) of the prelingual (pre) and postlingual (post) groups presented for the two stimulation electrode locations. The *box plots* represent the lower and upper quartile with the median. *Whiskers* indicate the 5–95 percentiles. *P < 0.05.

Grand averages of the eABRs evoked at apical and middle electrodes (Fig. 3) indicate that waves III of the two patient groups coincide, while wave V starts and peaks considerably later for the prelingually deaf patients when compared to the postlingually deaf. Accordingly, wave V latency was significantly longer in the nine prelingual subjects than in the nine postlingual subjects across electrode locations (*F*_(1,16)_ = 6.86, *P* = 0.019; Fig. 4A). Analyses for the two electrode locations separately revealed that on the apical electrode the average wave V latency in the 11 prelingual subjects was 3.9 ms, whereas in the postlingual subjects this was 3.6 ms (unpaired, two-tailed *t* test, *t*_*18*_ *=* 2.6, *P* = 0.020). On the middle electrode, wave V latencies were on average 3.9 ms for the prelingual subjects and 3.7 ms for the postlingual subjects (unpaired, two-tailed *t* test, *t*_*16*_ *=* 2.4, *P* = 0.030). If the three best performing prelingual users, pre 3, 5 and 11, were excluded from the analyses, wave V latency remained significantly longer than in the postlingual group (*F*_*(1,13)*_ = 13.48, *P* = 0.003).

Analysis of the wave III latency did not reveal differences between the prelingual group and the postlingual group (*F*_(1,16)_ = 1.14, *P* = 0.301). Average wave III latencies were around 2.0 ms in the prelingual group and 1.9 ms in the postlingual group on both electrode locations (apical electrode: unpaired, two-tailed *t* test, *t*_*18*_ *=* 1.1, *P* = 0.307; middle electrode unpaired, two-tailed *t* test, *t*_*16*_ *=* 1.2, *P* = 0.237). Wave III latency remained similar for both groups, if the three best performing prelingual subjects were excluded from the analyses (*F*_*(1,13)*_ = 1.71, *P* = 0.214).

The interwave III–V interval was longer for prelingually than for postlingually deafened, which was nearly significant when analyzed across both electrode locations (*F*_(1,16)_ = 4.43, *P* = 0.052). On the apical electrode the III–V interval was significantly longer in the prelingual subjects (average III–V interval prelingual group: 1.9 ms, postlingual group 1.7 ms; unpaired, two-tailed *t* test, *t*_*18*_ *=* 2.7, *P* = 0.015). On the middle electrode, the difference in III–V interval between groups was smaller and not significant (unpaired, two-tailed *t* test, *t*_*18*_ *=* 1.5, *P* = 0.143; Fig. 4B). If the three best performing prelingual users, pre 3, 5 and 11, were excluded from the analyses, the III–V interval in the prelingually deaf CI users, was significantly longer than in the postlingual subjects across both electrode locations (*F*_*(1,13)*_ = 7.05, *P* = 0.020).

### Factors Related to Wave V Latency and III–V Interval

**Fig. 5:**
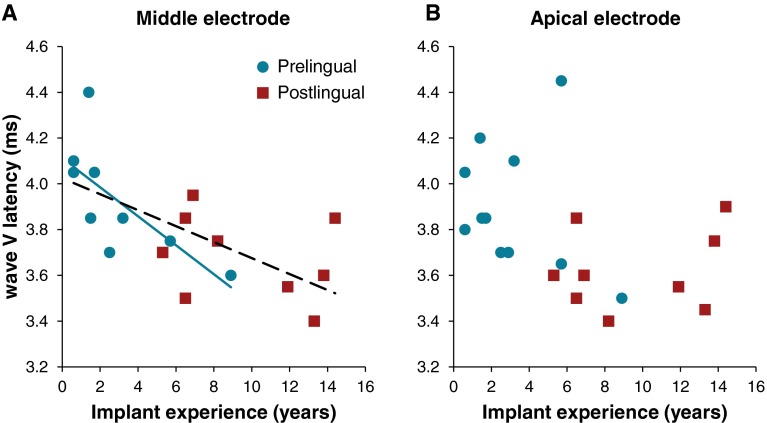
Wave V latency on the middle (*A*) and apical (*B*) electrode as a function of implant experience. Each *red square* represents a subject in the postlingual group. Each *blue dot* represents a subject in the prelingual group. On the middle electrode, there was a significant correlation between wave V latency and implant experience for the total group (*dotted line*, *r* = −0.665, *P* = 0.003, *n* = 18) and for the prelingually deaf subjects (*solid blue line*, *r* = −0.706, *P* = 0.034).

In order to assess if other factors, besides age at onset of deafness, could have contributed to the differences in wave V latency, stepwise multivariate regression analyses were performed. On the apical electrode, these analyses revealed that group was the only significant predictor of wave V latency (*r =* 0.516, *F*_*(1,18)*_ *=* 6.535, *P =* 0.020). Implant experience was not associated with wave V latency on this electrode (*P =* 0.846; Fig. 5B). Contrarily, implant experience was found to be the only significant predictor of wave V latency on the middle electrode (*r = −*0.665, *F*_(1,16)_ *=* 12.693, *P =* 0.003; Fig. 5A). Group was not a significant predictor of wave V latency on this electrode (*P* = 0.887). When examining both groups separately, wave V latency was significantly correlated to implant experience in the prelingual group (*r* = −0.706, *P* = 0.034; Fig. 5A), but not in the postlingual group (*r* = −0.324, *P* = 0.395). Previous studies on eABRs in children demonstrated that the wave V latency as function of CI experience could be best described by an exponential decay (Gordon et al. 2006; Thai-Van et al. 2007). If we describe the wave V latencies on the middle electrode in the prelingual subjects as an exponential, it would yield a time constant of about 4 years.

Wave V latency was not correlated to age at implantation on either tested electrode. Moreover, the other preoperative patient characteristics presented in Table 1 were not a factor determining wave V latency. Within the prelingual or postlingual group the wave V latency was not significantly correlated to age at onset of deafness.

The III–V interval was not associated with age at implantation, age at onset of deafness, or implant experience on either electrode location.

### Relationship Between eABRs and Speech Perception

As demonstrated in Figure 2, typical eABRs exhibiting both waves III and V could be recorded in almost all subjects irrespective of their speech perception scores or group (pre or postlingually deaf). Waveform morphology was however less clear in the prelingually deaf and poor performing subjects. Besides, in two prelingual subjects no clear eABRs could be recorded on the middle electrode (Fig. 2).

**FIG. 6:**
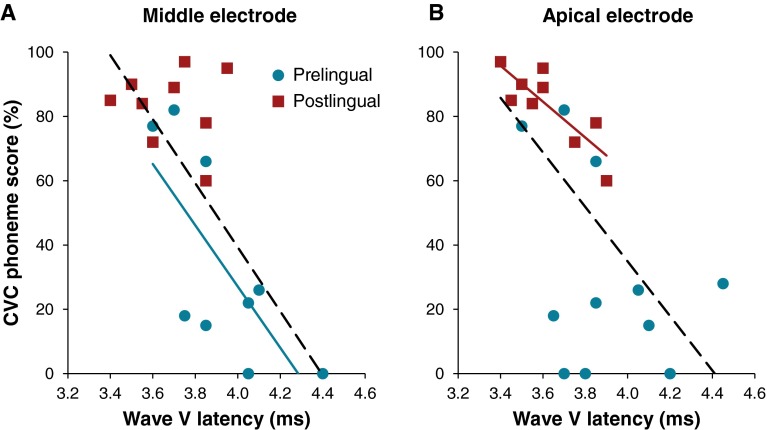
CVC phoneme score as a function of wave V latency for the middle (*A*) and apical (*B*) electrodes. Each *red square* represents a subject in the postlingual group. Each *blue dot* represents a subject in the prelingual group. The univariate analyses for the total group revealed significant correlations on both electrodes (*dotted lines*, Middle *r* = −0.706, *P* = 0.001; Apical: *r* = −0.640, *P* = 0.002). The *blue solid line* indicates a significant correlation within the prelingual group for the middle electrode (*r* = −0.728, *P* = 0.026), and the *red solid line* indicates a significant correlation within the postlingual group for the apical electrode (*r* = −0.833, *P* = 0.005).

Since postlingually deafened subjects show shorter latencies and better speech perception, a correlation is expected between these two measures. If all subjects were included in a linear regression analysis and if group was omitted, wave V latency was significantly associated with speech perception on both electrodes (Apical *r* = −0.640, *P* = 0.002; Middle *r* = −0.706, *P* = 0.001; Fig. 6 dotted lines). When the groups were analyzed separately a negative correlation between phoneme score and wave V latency was present on the apical electrode for the postlingual group (*r* = −0.833, *P* = 0.005; Fig. 6B solid line) and on the mid electrode for the prelingual group (*r* = −0.728, *P* = 0.026; Fig. 6A solid line).

Besides group, implant experience was significantly associated with speech perception (*r* = 0.580, *P* = 0.007). Stepwise multivariate regression analyses revealed that on the apical electrode wave V latency and implant experience were not predictive for speech perception, and only group remained a significant predictor (*r* = −0.755, *F*_(1,19)_ = 23.89, *P* < 0.001). On the middle electrode, group and wave V latency were significant predictors of speech perception explaining for 69 % of the variance (*r* = −0.828, *F*_(1,19)_ = 16.37, *P* < 0.001; variance explained by group: *37* %, *P* = 0.009 and by wave V latency: 32 %, *P* = 0.018). The III–V interval was significantly associated with postoperative speech perception when assessed over all subjects (Apical *r* = −0.632, *P* = 0.003; Middle *r* = −0.494, *P* = 0.037), but not when both groups were analyzed separately. Stepwise multivariate regression analyses confirmed that III–V interval was not a significant predictor besides group.

## DISCUSSION

In this study, we evaluated differences in auditory brainstem activation between pre- and postlingually deaf subjects who received a cochlear implant in adulthood. The extensive duration of auditory deprivation in the CI users with prelingual deafness appeared to have resulted in a delayed wave V while wave III was virtually unaffected. This delayed wave V activation may reflect a hindered neural and synaptic development especially in the more rostral part of the brainstem. To our knowledge, this is the first study to examine whether long durations of early onset auditory deprivation leads to delays in human auditory brainstem activation. These findings reveal that in the absence of auditory input throughout childhood, development of the rostral auditory brainstem seems to be hampered, due to degraded myelination and/or synaptic efficacy.

### Auditory Brainstem Development and Hearing Impairment

Neuropathological studies in autopsied fetuses and infants revealed that the auditory brainstem rapidly matures during the perinatal period. It is assumed that during the first two trimesters of pregnancy the anatomical structures involved in the auditory pathway are already developed, independent of sound-evoked activity (Moore and Linthicum 2007). The subsequent final maturation of the auditory brainstem is believed to result from sensory-driven processes of myelination, an increase in size of auditory neurons and rapid growth and branching of dendrites (Thai-Van et al. 2007; Moore and Linthicum 2007). Myelination of the auditory pathway starts around the 26th to 29th week and continues in the brainstem during the fetal and postnatal period (Inagaki et al. 1987; Moore et al. 1995; Moore and Linthicum 2007). By the age of 1 year, the myelin density of the cochlear nerve is comparable to that of an adult (Moore et al. 1995).

This myelination process and increase in synaptic efficacy of the auditory brainstem is probably reflected by the decreasing ABR wave latencies within the same period (Inagaki et al. 1987; Eggermont and Salamy 1988; Ponton et al. 1992, 1996). In the presence of normal auditory stimulation, ABR wave latencies mature over the first years of life (Inagaki et al. 1987; Eggermont and Salamy 1988; Ponton et al. 1992). In normal-hearing children, wave V latencies decrease following an exponential model, reaching adult values of 5–6 ms around the age of two. In the absence of normal auditory stimulation, congenitally deaf children show a significantly longer wave V latency when compared to children who became deaf after 1 to 4 years of age (Thai-Van et al. 2007). Among prelingually deaf children implanted between the ages of 1 to 17 years, the III–V wave interval recorded at activation of the CI does not depend on age at implantation (Gordon et al. 2006). In concordance with these findings, the results of our study show that after early onset of deafness and years of auditory deprivation, the wave V latency (and the wave III–V interval) is longer than in deaf subjects who experienced auditory stimulation in early development.

In children with early-onset deafness, it has been shown that wave V latencies decrease over the first years following cochlear implantation, in similar fashion as the exponential decrease with age observed in normal-hearing children (Gordon et al. 2006; Thai-Van et al. 2007). As suggested by our middle electrode recordings, duration of cochlear implant use may have been a factor contributing to the wave V latency in addition to onset of deafness (prelingual vs. postlingual). Fitting a decaying exponential function shows the time constant of this effect to be 4 years, which would be much longer than previously reported constants of 68 weeks (Thai-Van et al. 2007) or 5 months (Gordon et al. 2006) observed in children. Although not consistently found over both electrode locations, this effect of implant experience might suggest a possible sensory driven maturation of the auditory brainstem, which is still present even in adulthood after long durations of auditory deprivation. Acquiring longitudinal data will be necessary to clearly identify the role of chronic electrical stimulation in adults.

Animal models of deafness show that the major structures and pathways in the brainstem develop, but auditory input is required for the refinement of neuronal connections. Hence, in the absence of auditory stimulation, the cochlear nucleus becomes smaller and the projections received from the auditory nerve are broadened (Moore 1990; Leake et al. 2008), furthermore, cells shrink and synapses loose vesicles (Ryugo et al. 2010). The cochleotopic organization is however maintained despite deafness, but these broader connections might affect frequency resolution (Leake et al. 2008). In ferrets with bilateral cochlear ablation, the number of neurons projecting from the cochlear nucleus to the inferior colliculus was not different than that of normal-hearing animals (Moore 1990), suggesting that the coarse structure of the pathway is not affected by deafness. This is supported by a longitudinal study comparing eABRs of congenitally deaf cats with those with normal hearing (Tillein et al. 2012). This study showed that in the absence of auditory input, the normal eABR waves develop and wave latencies decrease at the same rate as in normal-hearing cats. Although wave latencies were comparable, eABR waveform morphology was less clear in the congenitally deaf cats. Moreover, the responses revealed a reduction in the amplitude of wave III and an increase of wave V amplitude (Tillein et al. 2012). The ongoing decrease of wave V latency in congenitally deaf cats after birth does not coincide with human data. This might be due to a discrepancy between the two species or due to a difference in stimulation (e.g., lower level in cats than in humans).

### Changes in Brainstem Responses and Cortical Potentials in Prelingually Deaf

Combining the results of the present study with the cortical potentials recorded in the same subjects (Lammers et al. 2015) gives an insight in the spontaneous development of the auditory pathway. In the prelingually deaf subjects, both the brainstem and cortical waveforms showed a normal morphology indicating that a coarse neural network from cochlea to cortex develops regardless of sensory input. Thus, even in the absence of sensory input throughout childhood, the development of this innate auditory pathway is not disrupted.

Remarkably, whereas the brainstem responses were delayed, the cortical responses in the prelingually deaf subjects showed significantly shorter latencies than the postlingually deaf subjects. We should stress that the cortical latency differences (~15 ms) are almost 100 times larger than the brainstem latency differences (~0.2 ms). Whereas myelination and synaptic development explain the relatively subtle ABR latency differences, other mechanisms play a role in cortical potentials. Sensory input representing biologically relevant sounds is crucial for refinement of the cortical network, in particular during childhood (Buonomano and Merzenich 1998; Innocenti and Price 2005; Ohl and Scheich 2005; Kral 2013). Thus, without such input, the cortex maintains a rather coarse organization containing fewer cortico-cortical connections with less top-down inhibition (Kral and Sharma 2012; Lammers et al. 2015). The different mechanisms of brainstem and cortex responses may also explain that eABR wave V latencies tend to decrease with implant experience, whereas such tendency is not found for CAEP latencies.

### Methodological Considerations

In our study population, a significant correlation between speech perception and wave V latency was found when eABRs were evoked at the middle electrode (Fig. 6). This agrees with a report by Gallégo et al. (1998) who also found an association between speech perception and wave V latencies. Although wave V latency on the middle electrode was found to be an independent predictor of speech perception, these results should be interpreted with caution. Larger patient series should be investigated to clarify the predictive role of eABR wave latencies, especially since there is no clear trend in the current literature studying relative small sample sizes (Abbas and Brown 1991; Brown et al. 1995; Gallégo et al. 1998; Makhdoum et al. 1998; Firszt et al. 2002; Kim et al. 2008; Gibson et al. 2009).

Variability among the CI patients was typically substantial. Notably, the speech perception scores among the patients classified as prelingually deaf varied widely (Fig. 1). Three of those subjects had high CVC scores and short wave V latencies, both characteristics being shared by postlingually deaf subjects. Since judgment of early onset of deafness is partly based on self-reported information (see “Methods” section), one cannot exclude significant use of residual hearing (also discussed in Lammers et al. 2015), which might have contributed to a better development of the brainstem and eventually to a better speech perception with their CI. On the other hand, sensitivity analyses excluding the three best performing prelingually deaf subjects did not change the found effects. Moreover, the early onset of deafness in these three subjects is supported by the cortical potentials which had latencies in the range of the other prelingually deaf subjects, significantly shorter than postlingual subjects (Lammers et al. 2015).

## CONCLUSION

Electrically evoked auditory brainstem responses in prelingually deaf late-implanted CI users demonstrate increased wave V latencies and interwave III–V intervals, suggesting delays in neural conduction within the auditory brainstem. These results indicate that long durations of hearing impairment directly or shortly after birth may lead to impaired neuronal connections within the innate and elementary parts of the auditory brainstem.
